# Le trichobezoard: une cause rare de masse abdominale

**DOI:** 10.11604/pamj.2014.17.31.2236

**Published:** 2014-01-18

**Authors:** Mohamed Rami, Youssef Bouabdallah

**Affiliations:** 1 Service de chirurgie pédiatique, CHU Hassan II, Fès

**Keywords:** Trichobezoard, masse abdominale, trichophagie, trichomanie, Trichobezoard, abdominal mass, trichophagia, trichomania

## Image en médicine

Le trichobezoard est une masse formée par l'accumulation des cheveux eu niveau du tube digestif, notamment dans l'estomac. Elle apparait chez la fille adolescente avec un profil psychologique particulier, présentant une trichomanie et une trichophagie. Nous rapportons le cas d'une patiente de 14 ans, qui consulte pour une masse abdominale sus ombilicale dure, indolore, fixe, mesurant environ 30 cm sur 14. La patiente et la famille niaient une trichophagie. La fibroscopie et le scanner sont revenus en faveur d'un trichobézoard. La patiente fut opérée par laparotomie, permettent l'extraction d'une masse de 1450 g.

**Figure 1:**
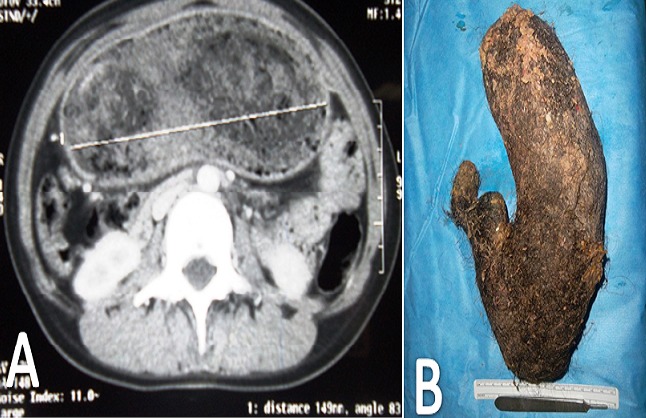
A) scanner abdominal montrant une masse hétérogène intragastrique sans anomalies pariétales; B) aspect de a pièce opératoire, masse poileuse de 1450 g, épousant la forme de l'estomac

